# Transcriptional bursting dynamics in gene expression

**DOI:** 10.3389/fgene.2024.1451461

**Published:** 2024-09-13

**Authors:** Qiuyu Zhang, Wenjie Cao, Jiaqi Wang, Yihao Yin, Rui Sun, Zunyi Tian, Yuhan Hu, Yalan Tan, Ben-gong Zhang

**Affiliations:** ^1^ Research Center of Nonlinear Sciences, School of Mathematical & Physical Sciences, Wuhan Textile University, Wu Han, China; ^2^ School of Mathematics, Sun Yat-sen University, Guangzhou, China; ^3^ School of Bioengineering & Health, Wuhan Textile University, Wu Han, China

**Keywords:** transcriptional bursting, single-cell sequencing data, data integration, gene expression model, parameter inference

## Abstract

Gene transcription is a stochastic process that occurs in all organisms. Transcriptional bursting, a critical molecular dynamics mechanism, creates significant heterogeneity in mRNA and protein levels. This heterogeneity drives cellular phenotypic diversity. Currently, the lack of a comprehensive quantitative model limits the research on transcriptional bursting. This review examines various gene expression models and compares their strengths and weaknesses to guide researchers in selecting the most suitable model for their research context. We also provide a detailed summary of the key metrics related to transcriptional bursting. We compared the temporal dynamics of transcriptional bursting across species and the molecular mechanisms influencing these bursts, and highlighted the spatiotemporal patterns of gene expression differences by utilizing metrics such as burst size and burst frequency. We summarized the strategies for modeling gene expression from both biostatistical and biochemical reaction network perspectives. Single-cell sequencing data and integrated multiomics approaches drive our exploration of cutting-edge trends in transcriptional bursting mechanisms. Moreover, we examined classical methods for parameter estimation that help capture dynamic parameters in gene expression data, assessing their merits and limitations to facilitate optimal parameter estimation. Our comprehensive summary and review of the current transcriptional burst dynamics theories provide deeper insights for promoting research on the nature of cell processes, cell fate determination, and cancer diagnosis.

## 1 Introduction

There are currently two primary recognized modes of gene expression: constitutive and bursty. Explosive transcription is a common occurrence in the human genome. In 2012, Dar et al. provided strong evidence of a theoretical framework for comparing gene expression patterns in cellular expression profiles (Dar et al., 2012). Transcriptional bursting represents a type of molecular dynamics that manifests as the heterogeneous expression of identical genes across different cells. The stochastic nature of transcriptional bursting and its potential for feedback regulation are integral to the maintenance of complex networks of biochemical interactions in living organisms.

An electron microscopy imaging study in the 1970s provided direct visual evidence of the discontinuous transcription of genes. Miller chromatin spreads from *Drosophila* embryos showed nascent transcripts distributed unequally along the gene sequence (Miller Jr and McKnight, 1979). The advent of fluorescence microscopy has advanced gene expression detection techniques such as single-molecule fluorescence *in situ* hybridization (smFISH) (Femino et al., 1998) and RNA phage MS2 stem-loop detection methods (Bertrand et al., 1998) on fixed cells. The studies in single cells have consistently recorded the rapid emergence and subsequent short-term disappearance of multiple mRNAs within a single gene (Raj et al., 2006; Battich et al., 2013; Larson et al., 2013; Lenstra et al., 2016). These imaging techniques not only confirm the discontinuity of transcription but also reveal transcriptional bursting that occurs on a timescale of minutes. In eukaryotes and prokaryotes, the dynamics of rapidly producing large amounts of mRNA in a short period is referred to as a “transcriptional burst”. Given the myriad life processes ongoing within an organism, cells continuously adjust their transcription processes to meet the demands of these activities. However, the internal dynamics of this process are complex (Tunnacliffe and Chubb, 2020). The study of rapid balance of gene states determines the state and function of discrete phenotypic cells, and feedback regulation significantly affects the switching of individual gene states (Ge et al., 2015). Ge et al. proposed a wave rate model to investigate the effect of random gene state switching dynamics by operons on the regulation of cell phenotypic specificity (Ge et al., 2018). Investigating the characteristics of transcriptional bursting, including the size and frequency of bursts and the degree of response to environmental, chemical, and genetic stimuli, we provide insights into the principles of transcriptional functions within the nucleus at the single-molecule level. Moreover, studies have reported that variations in the transcriptional bursting features can alter the cellular state (Fritzsch et al., 2018; Wang and Wang, 2021). Different cellular states can in turn influence the dynamics of transcription initiation and elongation (Mines et al., 2022). This is dependent on the interaction between internal and external noises, forming a rudimentary feedback loop in the regulation network. Transcriptional burst models of gene expression often discretize the continuous dynamics of gene expression into mathematical models of promoter switching. One of the earliest models used to describe gene expression was the protein synthesis model based on a Markovian framework (Peccoud and Ycart, 1995), known as the stochastic telegraph model of gene expression. This model characterizes the random switching of genes between the active and inactive states. With advancements in sequencing technology, the moment estimation method for inferring parameters in protein models has been improved to inferred mRNA models (Larsson et al., 2019). However, simplifying gene expression mechanisms by ignoring the complexity of intermediate states sacrifices many hidden stochastic molecular processes (Fritzsch et al., 2018). Therefore, Schwabe et al., Rodriguez et al., and Jia et al. independently developed multi-state models to map the mechanisms of sequence-encoded regulation on a genome-wide scale (Schwabe et al., 2012; Larsson et al., 2019; Jia and Grima, 2023). The steady-state solution of the analytical model reflects the dynamic equilibrium state of the gene expression system. In 2013, Kim et al. presented the Poisson-beta model based on the steady-state solution of the telegraph model, which was the first use of single-cell RNA sequencing (scRNA-seq) data (Kim and Marioni, 2013). In 2017, Jiang et al. proposed the SCALE framework to overcome ignoring technical variations in the model and attributed the source of noise in *PHO5* gene expression to nucleosome occupation and the differential expression of genes to the regulation of burst frequency (Jiang et al., 2017). In 2019, Larsson et al. inferred burst frequency and burst size from endogenous mouse and human genes using scRNA-seq data, which provided insights into how cis-regulatory sequences and transcriptional machinery govern these bursting characteristics (Larsson et al., 2019). Integrating transcriptional bursting with other factors or investigative methods is essential for the study of gene expression, regulation of gene activity, and the specificity of gene functions. In 2024, Wang et al. developed a comprehensive framework that integrated the dynamics of chromatin accessibility and transcriptional bursting (Wang et al., 2024). They enriched the theoretical modeling of gene expression mechanisms by constructing a stochastic gene expression model with feedback regulation. This model combines static promoter structures and dynamic regulatory networks using scRNA-seq data. In the same year, Fallacaro et al. quantified the molecular dynamics of transcription factor-specific hubs in *Drosophila* embryos using imaging technology and single-molecule tracking (Fallacaro et al., 2024). Their study showed that variations in burst duration, magnitude, and frequency control the different ways in which genes are expressed in the same cell nucleus (Fallacaro et al., 2024). Recently, Mayer et al. developed a gene expression model for multinucleated cells and showed that the division of transcriptional labor allows the syncytium to circumvent the tradeoff between gene expression efficiency and precision (Mayer et al., 2024). The stochastic nature of gene expression originates from regulation at different levels; intracellularly, multiple copies of the same gene can achieve similar developmental expression patterns during transcriptional bursting and receive distinct regulatory inputs for individual genes, a process that contributes to protein function diversity. Transcriptional bursting can affect expression states and behaviors by altering the communication between cells. This behavior results in the diversity and specificity of non-genetic transcription and sensitivity of cellular states to external interventions. Moreover, it affects how cells respond to the microenvironment and the modes of cell death. In emerging fields, such as spatiotemporal molecular medicine, transcriptional bursting provides novel insights into the molecular mechanisms underlying drug resistance (Wang and Wang, 2021).

The above studies either focused on specific medical directions without sufficient evidence or experimental results to support a unified conclusion (Rodriguez and Larson, 2020; Tunnacliffe and Chubb, 2020; Wang and Wang, 2021). The natural question is how to more comprehensively explain the stochasticity in the gene expression process using models or how to use advanced technology, such as scRNA-seq technology, to investigate the dynamics of transcriptional bursting in gene expression.

In this study, we provide a comprehensive review of the research contributions and current popular focus areas related to the mechanisms of transcriptional bursting, beginning with a systematic combination of various gene expression models and their applicability. Second, we elaborate on and summarize the key indicators of transcriptional bursting, the temporal scale separation of species-related mechanisms, and their degrees of impact. We then show the strategies for gene expression models from different perspectives and the philosophies, advantages, and disadvantages of classical parameter inference methods. Finally, we delve into the omics of single-cell data that drive the establishment of new gene expression mechanism models. We focus on the latest advancements and potential developments in the study of the mechanisms of transcriptional bursting dynamics. We further discuss the possible methods for exploring these dynamics and meaningful research directions. This study provides a comprehensive synthesis guidance for researchers in this field.

## 2 Models and methods

### 2.1 Gene expression model

In multicellular organisms, the configuration or compositional elements of promoters are crucial molecular mechanisms that determine transcriptional bursts. This is reflected in the allocation of promoter states and the stochastic pausing associated with the formation of specific biomolecular complexes at these promoters. The nucleosome in eukaryotes is the basic structural unit of chromatin, where the nucleosome is formed by combining DNA with histone proteins. The tight structure of chromatin leads to the silencing of genes, which is not conducive to transcription. During the slow opening of the DNA strand on the nucleosome, the gene will go through multiple deactivated states and eventually bind to transcriptional regulatory elements to activate transcription (Minnoye et al., 2021). At the transcriptional level, one way of the gene expression regulating is through induction fine-tuning, and an inducible gene is silenced most of the time. Such genes are briefly expressed when activated by external signals such as hormones, sugar and temperature. In order to model this biological process, Peccoud et al. first constructed model of the random switching of promoter states, which included the random switching of promoters between active and inactive states (Peccoud and Ycart, 1995). Two-state model of gene expression is commonly used as the random transcription model. However, for genome-wide studies, many experiments and theories have led to the development of multistep models to reflect the transcriptional dynamics and explain the heterogeneity of developmental gene expression. Here, we present the existing classical promoter state-switching models that represent one of the important advancements in understanding gene expression dynamics.

#### 2.1.1 One-state model

**FIGURE 1:**
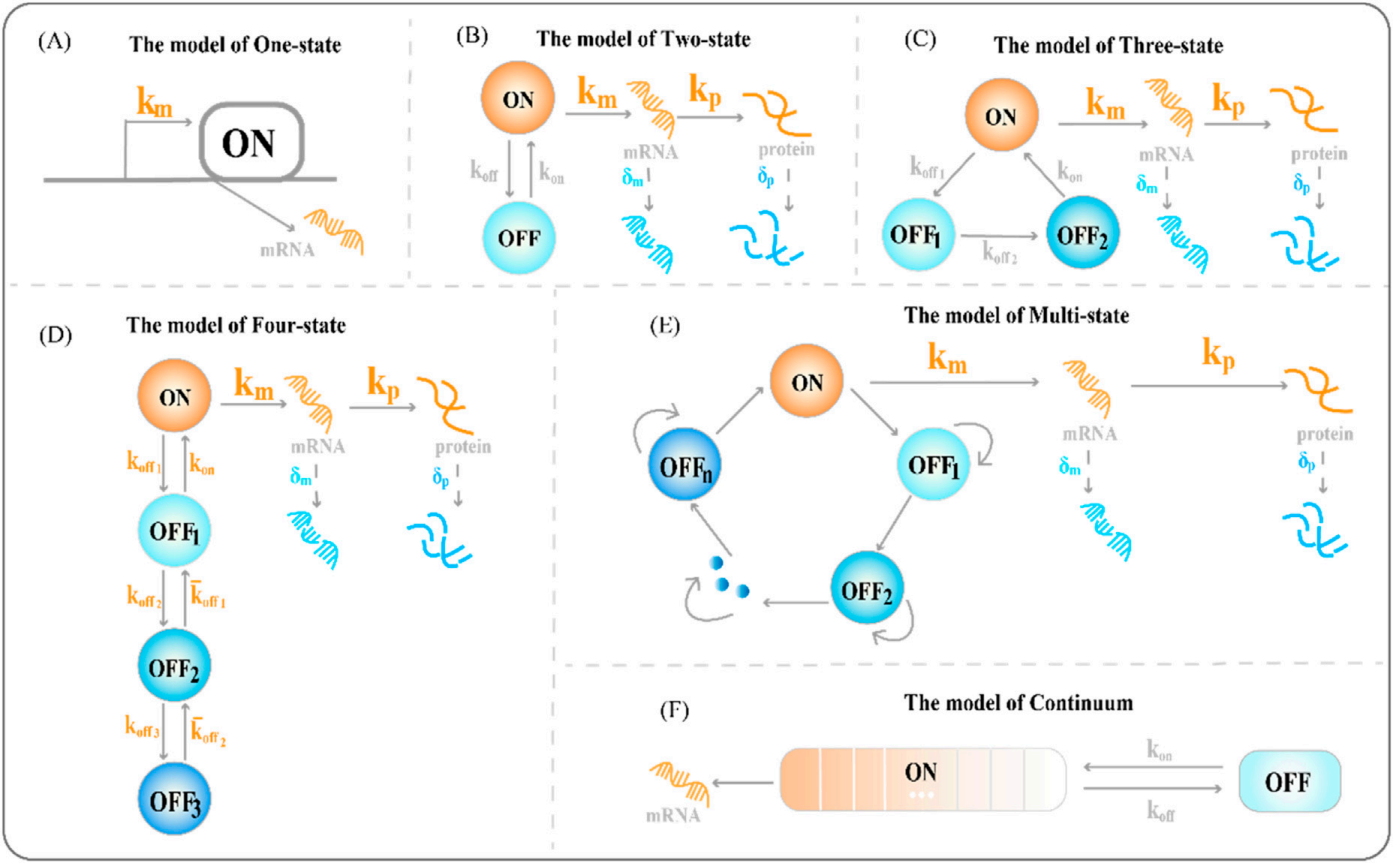
Classical transcriptional burst model of random gene expression **(A)** The one-state model of gene expression, where $k_{m}$ represents the transcription rate of RNA **(B)** The two-state model of gene expression (the random telegraph model), $k_{p}$ represents the translation rate of protein production, $k_{on}$ represents the rate of gene activation, $k_{off}$ represents the rate of gene inactivation, $\delta_{m}$ represents the rate of mRNA degradation, and $\delta_{p}$ represents the rate of protein degradation **(C)** The three-state model of gene expression includes the inactived state of two genes: the deeply inactived state $S_{off1}$ and the inactived state $S_{off2}$ **(D)** The classical chain four-state model of gene expression includes the inactived states of three genes: deep inactived state $S_{off1}$ , deep inactived state $S_{off2}$ , and inactived state $S_{off3}$ **(E)** The circular multi-state model of gene expression, consisting of multiple discrete gene inactived states and one activated state **(F)** The continuum model of gene expression, consisting of an actived state and an inactive state of a gene, and regulatory factors continuously regulating transcriptional initiation behavior over a long period.

To explore the source of heterogeneity in transcriptional dynamics and the most essential cause of random fluctuations, we need to understand the most basic one-state model of gene expression (the constitutive gene expression model). The one-state model describes the birth and death processes of gene products. Gene expression models describe genes that have multiple activated (ON) and inactivated (OFF) states: in the ON state, genes produce RNAs continuously at a constant rate, while existing RNA transcripts are degraded at a constant rate; in the OFF state, they stop producing RNAs, yet the degradation of existing RNA transcripts continues, as in the ON state. The one-state model of gene expression generally includes only a single active state $S_{on}$ (see Figure 1A) (Klindziuk and Kolomeisky, 2018), and some experiments have shown that the burst size of the gene follows a geometric distribution (Paulsson and Ehrenberg, 2000; Golding et al., 2005). At this point, the transcription process of the gene involves two simultaneous activities: the generation of RNA molecules at a constant rate and the degradation of RNA molecules at a constant rate. The rate is proportional to the number of existing RNA transcripts in the system. The one-state model consists of two effective reactions.

$$S_{on}\overset{k_{m}}{\to }S_{on}+mRNAs,$$

$$mRNAs\overset{\delta_{m}}{\to }\emptyset .$$ (1)

Single-molecule RNA fluorescence *in situ* hybridization (smFISH) can measure differences in RNA abundance and reveal differences between cells; however, the one-state model is not suitable for highly variable smFISH data (Nicolas et al., 2017). Furthermore, the steady-state distribution of the RNA molecules produced by the one-state model conforms to a Poisson distribution. However, this unique distribution of variance, equal to the mean, could not cover the transcriptional distribution of all genes and could not account for the overdispersion phenomenon in the results of the difference significance test of gene expression. Paulsson et al. found that adding additional gene expression states to the model could explain the differential expression phenomenon based on smFISH data (Paulsson, 2005).

#### 2.1.2 Two-state model

Studies on transcription in both prokaryotic and eukaryotic systems have yielded conflicting evidence about the primary modes of gene expression over extended periods. Several studies have focused on the elevated expression of mRNA alone (Golding et al., 2005; Taniguchi et al., 2010). By constructing a three-dimensional noise-space analysis framework, Dar et al. quantitatively analyzed the dynamic expression behavior of 8,000 gene loci and proved that the majority of human genomic loci appear to stochastically fire during episodic bursts. Combined with the high production rate and short activation time, bursting kinetics enable a more realistic distribution than previously studied one-state models (Herbach, 2019).

The two-state model of gene expression (see Figure 1B), is a phenomenological model that quantifies the burst dynamics of genes (Peccoud and Ycart, 1995). It does not require specifying the molecular identity of the burst parameters (Lammers et al., 2020). It can produce mRNA distributions of various shapes and mRNA copy numbers to reveal the underlying dynamics of the promoters. For example, short activation states cause long tails and high (hyperPoisson) variances in mRNA distributions; the slow promoter conversion rate and long waiting time for activated and inactivated states of the promoter cause two peaks in the mRNA distribution (Nicolas et al., 2017). The complete chemical reaction, based on the two-state model, is as follows:

$$S_{off}\overset{k_{on\;}}{\to }S_{on},S_{on}\overset{k_{off\;}}{\to }S_{off},$$

$$S_{on}\overset{k_{m}}{\to }S_{on}+mRNAs,mRNAs\overset{\delta_{m}}{\to }\emptyset ,$$

$$mRNAs\overset{k_{p}}{\to }mRNAs+proteins,proteins\overset{\delta_{p}}{\to }\emptyset .$$ (2)

The assumptions of the two-state model are brief but limited, and models containing multiple actived or inactived states are increasingly becoming alternatives to modeling transcription mechanisms that cannot be explained by them (Neuert et al., 2013; Bothma et al., 2014). This requires a careful balance between overfitting and predictive power of the model. Transcriptional burst behavior occurs on multiple timescales (see section 2.2 for details). The transcriptional burst gene expression models are a characterization of the degree of discretization of a continuous process in which chromatin compact structures are fully opened and fully closed. Promoter activity states have different fluctuations on multiple time scales; importantly, the transition between states may involve multiple rate-limiting steps, branching pathways, and molecular events (Tantale et al., 2016). However, the two-state model does not account for randomness.

#### 2.1.3 Three-state model

Genes produce mRNAs almost simultaneously at an unsteady rate, followed by a period of deactivation. Several models have focused on regulating parameters such as burst size and burst frequency to understand these dynamics (Brouwer and Lenstra, 2019). The two-state model largely fails to satisfactorily describe the transcription process. The two-state model assumes that burst decentralization is a secondary issue of transcription, occurring only because of specific internal molecular noise, and does not account for external sources of variation. In fact, chromatin opening is a slow process, and genes go through the refractory period before being activated again. Thus, a direct case for expanding the two-state model is to increase the refractory period (Suter et al., 2011a). The refractory period extends the two-state model to a three-state model (Figure 1C). The three-state model refines the long-occupied inactive state $S_{\text{off}}$ into a deeply inactive state $S_{off2}$ and an inactive state $S_{off1}$ . The complete chemical reaction, based on the three-state model, is as follows:

$$S_{off2}\overset{k_{on\;}}{\to }S_{on},S_{on}\overset{k_{off1\;}}{\to }S_{off1},S_{off1}\overset{k_{off2\;}}{\to }S_{off2}$$

$$S_{on}\overset{k_{m}}{\to }S_{on}+mRNAs,mRNAs\overset{\delta_{m}}{\to }\emptyset ,$$

$$mRNAs\overset{k_{p}}{\to }mRNAs+proteins,proteins\overset{\delta_{p}}{\to }\emptyset .$$ (3)

**FIGURE 2:**
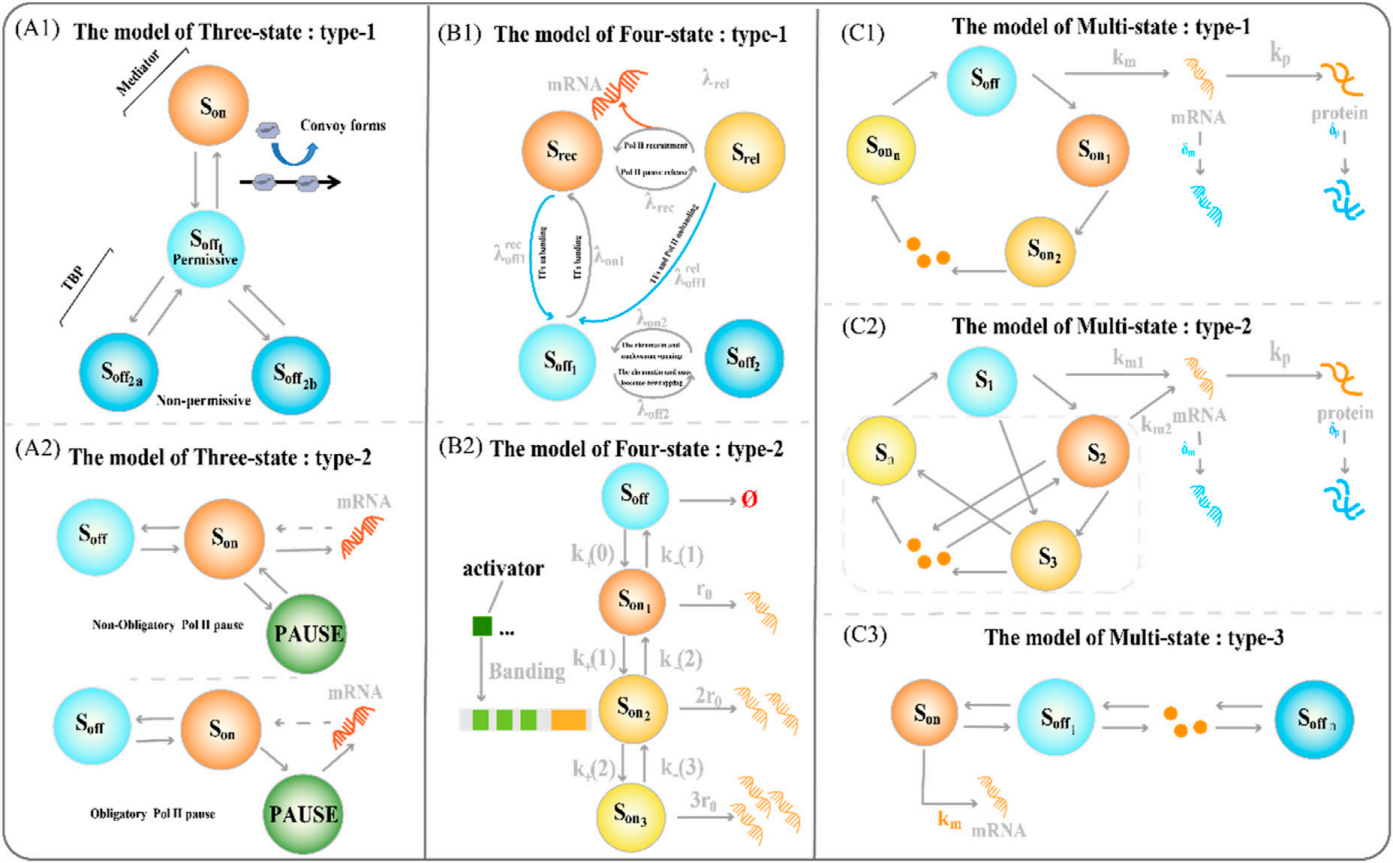
Classical transcriptional burst model of random gene expression **(A1)** The type-1 three-state model of gene expression. The regulation of the key factor TATA-box binding protein (TBP) determines the structure of the sub-OFF state **(A2)** The type 2 three-state model of gene expression type 2. The different degrees of RNA polymerase suspension shaped variants of the two three-state models **(B1)** The type-1 four-state model of gene expression integrates a framework that combines chromatin accessibility with transcriptional burst dynamics **(B2)** The type-2 four-state model type of gene expression consists of one inactive state and three activated states **(C1)** The type-1 multi-state model of gene expression consists of one inactive state and three activated states **(C2)** The type-2 circular multi-state model of gene expression consists of one activated state and several discrete inactivated states, with arbitrary connections between states **(C3)** The type-3 chain multi-state model of gene expression consists of one inactive state and several discrete sequentially activated states.

The latter allows a faster switch to the active state (Brouwer and Lenstra, 2019; Rodriguez et al., 2019). The three-state model also contains some variants (Figure 2(A1-A2)). The type-1 three-state model of gene expression (Figure 2A1) has multiple sub-OFF states, with the TATA-box binding protein (TBP) being a key protein and an important target for gene regulation (Tantale et al., 2016). An intermediate state appears when TBP is bound, and the long state appears when TBP is dissociated, encompassing the states of two non-licensing periods: $OFF_{2a}$ and $OFF_{2b}$ . The type 2 three-state model of gene expression type 2 (Figure 2A2), where RNA Polymerase II (RNA Pol II) pausing occurs on a minute timescale, is characterized by forced pausing (Pimmett et al., 2021). The three-state model with obligatory pause describes the systematic entry of all RNA Pol II molecules into a paused state, followed by extension and pause in mRNA production, while the three-state model with non-obligatory pause describes a random entry of a subset of RNA Pol II molecules into a paused state, followed by extension and pause in mRNA production.

#### 2.1.4 Four-state model

To refine the process of the slow opening of chromatin and capture important random events, we often need to add more states to accurately describe how static promoter sequences encode transcriptional burst dynamics. The classical four-state model of gene expression is a chain model (Figures 1D, 2B2) (Neuert et al., 2013; Rodriguez et al., 2019; Lammers et al., 2020). Furthermore, the integration of promoter-switching models with other dynamic models aims to uncover gene expression mechanisms that encompass more comprehensive information. Recently, Wang et al. addressed how upstream genomic spatial organization, particularly enhancer-promoter spatial communication, affects downstream transcriptional bursting dynamics by constructing a four-state model (Figure 2B1) (Wang et al., 2024). The OFF state of the model contains a deep inactive state ( $S_{off2}$ ) and an activated but inactive state ( $S_{off1}$ ), whereas the ON state of the model contains the Pol II recruitment state ( $S_{rec}$ (Fuda et al., 2009)) and Pol II suspended release state ( $S_{rel}$ (Chen et al., 2017)). Genes synthesize mRNA during the transition from state to state. The number of rate-limiting steps in gene transcription is usually small; therefore, the Markov’s four-state model can balance feasibility and efficiency. However, the context-specific four-state model is limited in its interpretation of transcriptional phenomena involving other molecular processes.

#### 2.1.5 Multi-state model

Early research on bacteria developed a series of *in vitro* single-molecule and live-cell experiments to model transcriptional bursting, where the waiting time between states of the two-state model follows an exponential distribution (Chong et al., 2014). However, there is a “refractory” behavior in genes transcription of mammalian cells that produces a distribution of non-exponential peaks (Suter et al., 2011a). Promoters with multiple activation states also exist, resulting in a non-exponential distribution of activated state wait times (Sepúlveda et al., 2016). Corrigan et al. found that continuously varying activation states can describe experimental data more accurately than discrete states, enabling a wide dynamic range of cellular responses to stimuli (Corrigan et al., 2016). The “refractory” behavior in this gene reflects molecular memory, and the existence of molecular memory in different states further affects the differential peak shape of gene product number distribution. In fact, molecular memory is a feedback mechanism for inducing bimodal, fine-tuning expression noise, and inducing promoter-switching memory (Zhang and Zhou, 2019). Molecular memory is a non-Markov process that simulates reactions within cells, helping to identify more molecular details of biological processes. The previous model reduced the non-Markov problem to a Markov problem without considering that the switching of gene states between active and inactive states is a multi-step process. At present, there have been studies to reveal the non-Markov properties of kinetics by modeling the waiting time of each state as a non-exponential distribution. We introduce these models in the following sections.

##### 2.1.5.1 Typical multi-state model

Understanding how multiple interacting elements cause genes to switch randomly between different depths of active states during transcription is crucial. Therefore, we constructed a multi-state model of gene expression to study the dynamics of transcriptional bursts. A chain multi-state model is the first to explain this complex promoter-switching mechanism (Figure 2C3). Previous studies focused on the steady-state behavior of systems. The steady-state distribution of mRNA copy numbers usually satisfies the generalized hypergeometric functions. A natural choice is to extend the chain multi-state model to a ring model with one active and multiple inactive gene states (Figure 1E).

From the perspective of the transient behavior of the system, Jia et al. studied the time-dependent distribution of mRNA and protein copy number (Jia and Li, 2023). They integrated multiple promoter configurations to establish a gene expression model that described complex promoter switching. The complete chemical reactions based on this model are as follows:

$$S_{i}\overset{k_{ij}}{\to }S_{j},i,j=0,1,2,\cdots ,L,i\neq j,$$

$$S_{0}\overset{{\;\varphi }_{0}p^{k}q\;}{\to }S_{0}+kp,S_{i}\overset{{\;\varphi }_{1}p^{k}q}{\to }S_{j}+kp,k\geq 0,$$

$$p\overset{\delta }{\to }\emptyset .$$ (4)

Where the parameter follows a geometric distribution, q=1−p . The first line of reaction in the system indicates that the promoter switches between all gene states at the rate $k_{ij}$ ; the second line of reaction describes the generation of gene product p in all gene states. When k=0 , the generation of gene product p is constitutive, and when k≥1 , the generation of gene product p is bursty. The last line of reactant shows that the gene product p decays at rate δ . The rate $\varphi_{0}p^{k}q$ and $\varphi_{1}p^{k}q$ indicate the reaction tendency functions and describe the state switch of the promoter. When the promoter is in the $S_{0}$ state, the gene product is produced at rate $\varphi_{0}$ , and when the promoter is in the $S_{i}$ state, the gene product p is produced at rate $\varphi_{1}$ . When $\varphi_{0}<\varphi_{1}$ , $S_{0}$ is active state, $S_{i}$ , i=1,2,⋯,L is inactive. In this case, the active period presents an exponential distribution, and the corresponding model is called the multiple OFF states model (Figure 2C1), and the inactive period may present a non-exponential distribution. When $\varphi_{0}>\varphi_{1}$ is inactive, $S_{0}$ , i=1,2,⋯,L , is the active state, the active period is non-exponential distribution, and the corresponding model is called the multiple ON states model (Figure 2C2). Dividing multi-state models directly based on the activation state of genes is a simple and direct approach. Some studies categorize multi-state models according to the waiting time of the activation state (Daigle et al., 2015).

##### 2.1.5.2 Continuum model

The snapshot data obtained from the population of dead cells via scRNA-seq did not allow the observation and quantification of the continuous evolution of transcriptional behavior over time (Chubb et al., 2006). A quantitative imaging study of actin gene transcription revealed that its activity of gene transcription is not strictly discrete, but resembles a continuous or dynamic spectrum of states (Corrigan et al., 2016). This suggests that there is a wide dynamic range of cellular responses to stimulation. Corrigan et al. constructed a continuum model of gene expression based on the two-state burst model (Corrigan et al., 2016). This model includes a long-term inactivated state of the gene and the activation state of genes that continuously switch due to slow fluctuations in the activation rate (Figure 1F). The continuum model simulates suitable dynamic gene expression data for the immediate response of cells to stimuli (Featherstone et al., 2015) and provides a suitable scenario for interpreting the continuous output of transcriptional products (Sepúlveda et al., 2016). The study found that most promoters have more than two effective states (Harper et al., 2011; Zhang et al., 2012). In the multi-state model, transitions between switching states expand the reaction steps to describe more complex transcriptional burst regulation mechanisms (Schwabe et al., 2012).

#### 2.1.6 Comparison of models

The telegraph model first rigorously links transcriptional dynamics to random gene expression. Researchers commonly use the two-state model for gene expression studies owing to the conciseness of its assumptions. The simplicity of this model stems largely from the assumption of constant rates during gene state switching and transcription. Morepver, these assumptions attribute burst behavior to internal molecular noise, without accounting for the influence of external variant signals. In particular, the two-state model is insufficient to explain the dynamic process of eukaryotic gene transcription that involves a large number of regulatory proteins and cofactors (Schwabe et al., 2012). Earlier reports have added to the bias between the refractory period and the two-state model; the bias extends to the three-state model, which includes refined periods of activity and inactivity (Suter et al., 2011a). The advantage of this model is in its ability to explain the control of explosive mRNA production and is suitable for genome-wide studies. However, it may lead inefficient information transfer in multi-state transcription. Models with only one or two gene states cannot accurately describe the dynamic transcription of several genes. Multi-state models can avoid these limitations of and offer a more accurate depiction of gene expression dynamics (Elgart et al., 2011; Schwabe et al., 2012; Zhang and Zhou, 2014; Zoller et al., 2015; Livingston et al., 2023; Wang et al., 2023; Chen et al., 2024). However, researchers have guided the design of the model based on prior knowledge of the system, specific research objectives, and the subjects limiting it. Table 1 summarizes the advantages and disadvantages of the various gene expression models.

**TABLE 1:** Summary and comparison of random gene expression models, where BCR is biochemical reaction.

Model	Bcr	Advantage	Disadvantage	Ref.
One-State Model	Equation 1	A fixed initiation rate	Lack of complexity Overdispersion	Klindziuk and Kolomeisky (2018)
Two-State Model	Equation 2	Widely applicable Distribution BS Steady-state distribution of mRNA.	Constant rate Lack of randomness No external noise	Peccoud and Ycart (1995), Lammers et al. (2020)
Three-State Model	Equation 3	Controlled burst synthesis Genome-wide applicability	Inefficiency of information transfer	Suter et al. (2011a), Tantale et al. (2016), Pimmett et al. (2021)
			Lack of complexity	
Multi-State Model	Equation 4	Gene regulation High flexibility	Clear object of study	Daigle et al. (2015), Corrigan et al. (2016), Chen et al. (2024)
			Specific prior assumption	

Overall, using a two-state model to simplify the description of a gene’s transcriptional burst should not be the default approach; likewise, some form of multi-state structure is not guaranteed to be more descriptive (Nicolas et al., 2017). Therefore, we must choose the most suitable model that can best reveal the nature of the differentiation destiny of a certain organism according to various data types to achieve a better match between the theoretical model and experimental data. In addition, the gene expression models we constructed were all independent models based on specific problem situations, with a large number of models and varying degrees of complexity. Therefore, biological processes in complex biological systems require the integration of multiple models for regulation. Random telegraph model can be used as the basic unit to construct interaction gene regulatory networks (Herbach et al., 2017).

Traditional multi-state models of gene expression lack the ability to capture randomness affecting observed mRNA numbers, such as the inability of telegraph models to account for the effects of external noise on gene transcription and switching rates. Fortunately, several studies have addresssed this by modeling the effects of noise on mRNA and protein abundances, that can affect parameter estimates, from data and model driven perspectives, respectively. From a model perspective, Durrieu et al. coupled gene expression to cell size and cell-specific nuclei (Durrieu et al., 2023); Jia et al. and Wang et al. correlated polymorphic models of gene expression with cell size, cell cycle stage, and gene dosage compensation coupling (Jia and Grima, 2023) (Wang et al., n.d.); and Thomas et al. coupled gene expression with cell division and cell differentiation (Thomas and Shahrezaei, 2021). From a data perspective, Tang et al. considered the effects of cell size and counting noise on gene expression (Tang et al., 2023), and Grima et al. identified the source of external noise in gene expression based on parameter deviation characteristics (Grima and Esmenjaud, 2024). In conclusion, focusing on improving model randomness in gene expression research is more in line with the complexity of biochemical systems.

### 2.2 Molecular mechanism of transcriptional burst and its regulatory factors

#### 2.2.1 Time scale of transcriptional burst mechanism

Bacteria, as representative prokaryotes, and mammalian cells, as representative eukaryotes, intermittently produce transcripts on different timescales. The synthesis and processing of these products involve molecular mechanisms across multiple scales of time. The experimental results of gene expression measurements at different time resolutions, ranging from milliseconds to days (Harper et al., 2011; Fritzsch et al., 2018; Rodriguez et al., 2019), provide a basis for understanding gene regulation mechanisms.

**FIGURE 3:**
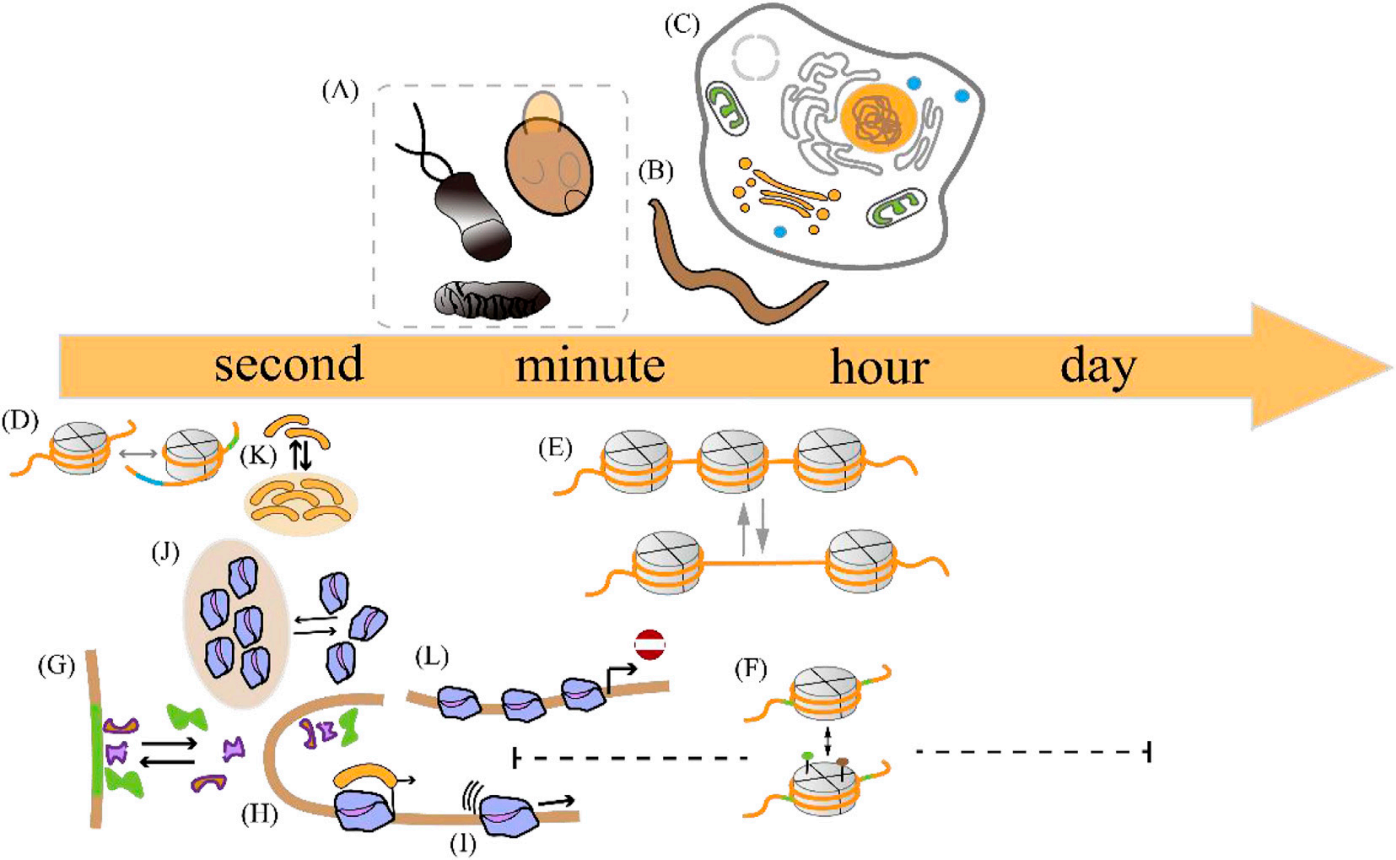
Temporal scale separation of transcriptional bursting and its underlying molecular processes across different species. The time scales of transcriptional bursts at different species levels are shown above the time scale arrows. The time scales of potential molecular processes associated with transcriptional bursts are shown below the time scale arrows **(A)** Transcriptional bursts in an embryo of the fruit fly *Drosophila melanogaster*, yeast, and bacteria occur on timescales of a few minutes **(B)** Transcriptional bursts in the nematode *Caenorhabditis elegans* occur on timescales of minutes to hours **(C)** Transcriptional bursts in human cells occur on timescales of a few hours **(D)** The process of DNA wrapping or unwrapping of nucleosomes occurs on timescales of milliseconds **(E)** Nucleosome turnover in the chromatin of eukaryotic cells occurs on timescales of minutes to hours **(F)** Histone modification occurs on timescales of minutes to days **(G)** Transcription factor binding occurs on timescales of a few seconds **(H–I)** Enhancer-promoter interaction and transcription initiation occur on timescales of seconds to minutes **(J)** RNA polymerase cluster kinetics occurs on timescales of seconds to minutes **(K)** Media cluster dynamics occur on timescales of seconds to minutes **(L)** The promoter-proximal pausing behavior occurs on timescales of seconds to minutes. The details are shown in Table 2.

The duration of a transcriptional burst is the sum of the time course during which multiple transcription initiation events occur (Wang et al., 2018). Despite advances in imaging techniques and single-cell sequencing, the accurate measurement of the duration of transcriptional bursts remains challenging. The existence of multiple time scales Figure 3 can be explained by variations in the duration of individual transcriptional bursts across different organisms and genes (Figure 3 (1–3), Table 2 and 3) and variations in the temporal resolution of different underlying molecular processes related to transcription (Figure 3 (4–11) and Table 4). Experiments by Pichon et al. on the molecular activity of the TATA-binding protein (TBP) and the pre-initiation complex (PIC) revealed the following three timescales of promoter activation in steady-state systems (Pichon et al., 2018): (1) long inactive periods and brief active periods, (2) 1-min transcriptional intervals produced by TBP binding, and (3) faster fluctuations between active and inactive promoter states induced by TBP binding and subsequent molecular activities. It is critical to determine the timescales of the transcription process and build interpretable and analyzable mathematical models of promoter states. Different genes display distinct bursting characteristics in biological processes with different timescales, and regulation of burst size influences the degree to which cells respond to stimuli and the extent of variability in downstream gene products.

**TABLE 2:** Time separation of single transcriptional bursts in different biological systems.

Bio-system	Bacteria	Fruit fly embryo	Nematode	Human, mouse	Amoeba
Gene	*In vitro* Tet system	even-skipped Notch signaling; gap genes; hunchback	Notch signaling	TGF-β Signaling TFF-1 signaling; liver genes; Mammalian genes	actin gene family 1 actin gene family 2
Timescale	minutes	minutes	minutes hours	hours	minutes hours
Ref.	Chong et al. (2014)	Bothma et al. (2014), Desponds et al. (2016), Falo-Sanjuan et al. (2018), Lammers et al. (2018), Zoller et al. (2018), Berrocal et al. (2020)	Lee et al. (2019)	Suter et al. (2011a), Molina et al. (2013), Bahar Halpern et al. (2015)	Corrigan et al. (2016), Tunnacliffe et al. (2018)

**TABLE 3:** Time isolation of potential transcriptional molecular mechanisms in eukaryotes.

Molecular processes	Nucleosomal DNA wrapping/unfolding	Nucleosome Turnover	Histone modification
Regulatory molecule	Mononucleosomes Mononucleosomes Mononucleosomes	Histone H3.3 Histone H3 Histone H1,H2B,H3and H4 tagged with GFP	dCas9 inducible recruitment rTet Rinducible recruitment Histone H3 Targeted recruitment; liver genes; Histone H2a, H2b, H3, H4 Histone H2, H2a, H2b
Organism	*In vitro* reconstitution	Fruit fly cell Yeast, Plant cell Human cell	Mammalian cell Human cell Yeast Mammalian cell
Times-scale	Milliseconds Seconds	Hours Minutes	Day Hours Minutes
Ref.	Kassabov et al. (2003), Li et al. (2004), Tomschik et al. (2005)	Waterborg (1993), Misteli et al. (2000), Kimura and Cook (2001), Dion et al. (2007), Deal et al. (2010)	Chestier and Yaniv (1979), Katan-Khaykovich and Struhl (2002), Zee et al. (2010), Hathaway et al. (2012), Bintu, 2016; Braun et al. (2017)

**TABLE 4:** Molecular regulatory mechanisms affecting transcriptional bursts in higher eukaryotic genes, as depicted in Figure 5.

Molecular mechanism	Influence index (BS/BS&BF/BF)	The index affects the crude proportion (BS/BS&BF/BF)	Ref.
Local chromatin environment	BS/BF	50%/37.5%/12.5%	Singh et al. (2010), Skupsky et al. (2010), Larson et al. (2013), Singer et al. (2014), Zoller et al. (2015), Fukaya et al. (2016), Mazzocca et al. (2021)
Nuclesome occupancy	BF	1	Dey et al. (2015)
Histone modifiaction	BS/BS&BF/BF	57.1%/14.3%/28.6%	Muramoto et al. (2010), Harper et al. (2011), Suter et al. (2011a), Dar et al. (2012), Viñuelas et al. (2013)
Number of cis-regulator elements	BS	1	Raj et al. (2006), Suter et al. (2011a), Viñuelas et al. (2013)
Affinity of cis-regulator elements	BS/BS&BF	50%/50%/0	Suter et al. (2011a), Corrigan et al. (2016)
DNA looping	BS&BF/BF	0/33.3%/66.7%	Bartman et al. (2016), Fukaya et al. (2016)
Transcription factors availability	BS/BS&BF/BF	13.3%/33.3%/53.4%	Raj et al. (2006), Singh et al. (2010), Suter et al. (2011a), Dar et al. (2012), 2016; Larson et al. (2013), Ochiai et al. (2014), Senecal et al. (2014), Singer et al. (2014), Bahar Halpern et al. (2015), Kalo et al. (2015), Xu et al. (2015), Ezer et al. (2016), Kafri et al. (2016)

Burst size (BS): the number of copies transferred by a transcription burst.

Burst frequency (BF): the number of transcriptional bursts that occur in a fixed cycle.

Within a single gene, these multiscale transcriptional bursts can occur independently and simultaneously (Tantale et al., 2016), encompassing the complex dynamics of bursting behavior. Recently, several studies have focused on extending the traditional two-state model to include additional insights and validations of these stochastic processes (see Table 4), while preserving the tractability of the model analysis (Kim and Marioni, 2013; Vu et al., 2016; Larsson et al., 2019; Chen et al., 2022; Grima and Esmenjaud, 2024).

#### 2.2.2 Regulatory factors affecting transcriptional burst mechanism

Cells with the same genome in a common environment exhibit heterogeneity in gene expression, which is reflected in expression patterns and degrees of expression. In eukaryotic organisms, a greater degree of gene-specific behavior in gene expression relies on the description of burst characteristics, with burst size and burst frequency commonly used to analyze the mechanisms of transcriptional bursting. These burst features are regulated by molecular mechanisms such as the local chromatin environment, nuclear occupancy, histone modifications, number and affinity of cis-regulatory elements, DNA looping, and transcription factors (see Figure 1). To understand the extent to which these molecular mechanisms drive burst dynamics, we analyzed the influence of molecular mechanisms on burst size and frequency, drawing from a comprehensive body of literature that quantitatively assesses the impact of regulatory factors in various gene expression contexts (Table 4). The degree of influence on the burst indicators was proportionally delineated based on the referenced volume in the literature. The number of cis-regulatory elements exclusively affects burst size (Dey et al., 2015). However, nuclear occupancy, particularly at transcription termination sites (TTS), dominates the regulation of burst frequency and it acts as a key factor driving burst dynamics (Raj et al., 2006; Suter et al., 2011b; Senecal et al., 2014). Histone modifications and the affinity of cis-regulatory elements primarily influence burst size, whereas DNA looping and the acquisition of transcription factors mainly affect burst frequency. A study based on the two-state model revealed that the local concentration of transcription factors around a gene and their residence time at the binding sites jointly regulate the size and duration of transcriptional bursts (Suter et al., 2011a). However, the relationship between the activity of target genes and the binding rates of transcription factors is being further investigated (Ochiai et al., 2020), as the correlation between transcription factor binding rates and transcriptional burst frequency is not universally observed (Rullan et al., 2018). In addition, other cellular factors may play a role in the regulation of transcriptional dynamics (Lenstra et al., 2016), such as the regulation of burst frequency by promoter-enhancer proximity during dominant developmental processes (AntoloviÄ‡ et al., 2017; Alexander et al., 2018; Chen et al., 2018).

### 2.3 Research methods for gene expression models

We divided existing analysis and solution methods of previously reported gene expression models into two categories: the biostatistical perspective and the biochemical reaction network perspective. To capture the randomness of transcriptional burst mechanisms in gene expression, models usually assume that all biochemical random events are Markov processes, considering the ease of the model. From the perspective of biostatistics, the best way to characterize the randomness of gene products is through a probability distribution. The goal of the researchers is to fit ideally the steady-state distribution predicted by gene expression models with the count distribution of single-cell mRNA snapshots generated by sequencing techniques, such as smFISH or scRNA-seq. From the perspective of the master equation, a biochemical reaction network describes the dynamic changes in the state of a transcriptional burst biochemical system. It is a group of differential equations that describes the probability density function of the change in the number of species in a biochemical system. The appropriate choice of statistical tools helps gain finer insights from the same transcriptome data.

#### 2.3.1 Biostatistical perspective

From a traditional biostatistical perspective, transcription is described as a low-probability event. In eukaryotic cells, transcriptional products are generally considered to be generated by the Poisson process, based on the classical telegraph model of gene expression. In the case of bursts, a negative binomial distribution is considered to be the canonical distribution of intracellular dynamics. When the introduced technical noise and biological noise are considered, the mRNA counts are divided into real counts and observed counts, and the mRNA counts of the telegraph model are often characterized by a mixed distribution (Wang et al., 2018; Luo et al., 2022a). Based on the results of cell-sequencing experiments, we often need to assume the probability distribution of the observed sequencing counts to supplement the analysis before modeling the gene expression mechanism. The authenticity of the biological processes and their rationality must be considered. The count data for gene expression are generally discretized, and discrete distribution is best used to describe such data, excluding cases where the entire number of expression counts is extremely high (Amrhein et al., 2019).

##### 2.3.1.1 Poisson distribution

Poisson distribution is prevalent in various random dynamics of gene expression, regardless of whether it is time-dependent or stationary. The Poisson distribution describes the number of independent events within a given period. In systems biology, the Poisson distribution variable represents the number of independent events that produce a biomolecule. The Poisson distribution was derived from a simple gene expression one-state birth-and-death model. The Poisson process describes the number of generated events in the time interval of transcription bursts, which represents the lifetime of a molecule destined to survive for a given duration. The negative binomial distribution of mRNA counts occurs as a steady-state distribution derived from a kinetic model that produces mRNA molecules in a burst form (Amrhein et al., 2019). It assumes a convenient tradeoff between computational complexity and biological simplicity.

##### 2.3.1.2 Negative binomial distribution

Most mammalian genes are described using transcriptional burst models of gene expression, which implicitly serve as the basis for a negative binomial model of scRNA-seq counting (Gorin and Pachter, 2020). The negative binomial distribution is itself a distribution of discrete random variables that describe the probability of the number of failed events X observed in a series of independent Bernoulli experiments until a predefined number of successes occurs, that is $∼NB\left(r,p\right)$ , where the success probability is $p\in \left[0,1\right]$ and the predefined number of successes is $p\in \left[0,1\right]$ . The probability quality function of the number of failed events X is Equation 5

$$f_{NB}\left(x;r,p\right)=P_{NB\left(r,p\right)}\left(X=x\right)=\left(\begin{array}{c} x+r-1\\ x \end{array}\right)p^{r}{\left(1-p\right)}^{x},for\;x\in N_{0}$$ (5)

In early studies of gene expression, the negative binomial (NB) distribution was considered suitable for random gene expression models in mathematics, but there was no adequate explanation in biology (Raj et al., 2006). In 2019, Amrhein et al. combined stochastic differential equations and the chemical master equation (CME) to build an interpretable mechanism model that could directly derive the NB distribution under steady-state conditions (Amrhein et al., 2019). In the parameter setting, they regarded transcription events as failure events and gene inactivation events as success events. In gene expression models that include splicing dynamics, the NB distribution exists as a marginal distribution containing the combined distribution of nascent and mature mRNA counts, which helps fit the observed single-cell data (Gorin et al., 2021). The solution of the telegraph model in the burst limit can be approximated as a negative binomial distribution (Paulsson and Ehrenberg, 2000) or a zero-expansion NB distribution, such as the three-state model of gene expression (Jia, 2020). Of course, it is difficult to directly obtain a mechanistic model of NB distribution under a steady state. Therefore, continuous Poisson-beta and Poisson-gamma distributions are common methods for inferring the evolution process. The Poisson-beta, Poisson-gamma, and NB distributions are mathematically equivalent.

##### 2.3.1.3 Poisson-beta distribution

Based on the classical telegraph model of random gene expression, when all aspects of biological variation and technical noise, including cell size and dropout rate, are considered, the mRNA count is divided into two types: real and observed counts. Owing to the overly decentralized nature of gene expression data, a common statistical method is to use the Poisson-beta distribution with three parameters (Kim and Marioni, 2013; Tang et al., 2018; 2023). The mRNA count distribution $X_{ij}$ of the two-state model follows a mixed distribution as follows in Equation 6:

$$X_{ij}∼Poisson\left(kp_{i}\right),$$

$$p_{i}∼Beta\left(\alpha ,\beta \right).$$ (6)

The random variable $X_{ij}$ is the mRNA count observed in the j gene of the i cell, following the Poisson distribution; its parameter $kp_{i}$ , $p_{i}$ is the original mRNA count in the i cell, that is, the original count follows the distribution, and k is the effective transcription rate acting on the original mRNA count. The observed mRNA count $X_{ij}$ follows a Poisson-beta distribution. Although this distribution provides good results for the estimation of RNA-seq data, it has a high computational cost because of its large number of parameters.

##### 2.3.1.4 Poisson-gamma distribution

The mRNA count distribution has been modeled based on the two-state model of gene expression. Some studies suggest that the observed count of gene expression levels in cells under steady-state conditions follows a conditional probability distribution that adheres to a Poisson distribution, and the promoter switching dynamics obey a gamma distribution. When all aspects of biological variation and technical noise are factored, the mRNA counts of the two-state model are follow a mixed distribution: the Poisson-gamma distribution like Equation 7 (Amrhein et al., 2019).

$$X_{ij}∼Poisson\left(kp_{i}\right),$$

$$p_{i}∼Gamma\left(\alpha ,\beta \right).$$ (7)

#### 2.3.2 Biochemical reaction network perspective

The complexity of the gene expression process implies that it involves numerous biochemical reactions, which reduce the process to a set of biochemical reaction networks (including reaction rates) after coarse-grained processing of molecular details (T, 2019) seeing Equation 8.

$$\sum_{j=1}^{n}r_{ij}X_{j}\overset{k_{i}}{\to }\sum_{j=1}^{n}s_{ij}X_{j},1\leq i\leq M,1\leq j\leq n,$$ (8)

where $r_{ij}$ and $s_{ij}$ are stoichiometric, that is, the amount of change in the number of molecules of species $X_{j}$ with reference to the i th reaction and $k_{i}$ is the reaction rate, such as the transfer network between the promoter states. The CME is not only the basis for establishing a gene expression model but also contributes to the dynamic change of the biochemical system state. The differential equation describing the probability density function $P\left(X;t\right)$ of the change in the number of species molecules X with time t in a biochemical reaction (T, 2019) is written as follows Equation 9:

$$\frac{\partial P\left(X;t\right)}{\partial t}=\sum_{i=1}^{M}\left(E^{-v_{i}}-I\right)\left[a_{i}\left(X\right)P\left(X;t\right)\right],$$ (9)

where I represents the identity operator, E and its inverse $E^{-}$ are translation operators, and $a_{i}\left(X\right)$ is the reaction tendency function. Our aim is to construct meaningful and interpretable CMEs, especially selecting the right solution tools, which are the key to studying gene expression models, and finally obtain a steady-state solution and determine the statistical significance of the distribution that the variables follow.

Currently, methods based on simulation, matrices, and analysis are the three most common methods used to solve the main equations of chemistry. Simulation-based methods, such as the well-known Gillespie stochastic simulation algorithm (Gillespie, 1976; 1977), are used to solve difficult to find analytical solutions to the CME. The Gillespie algorithm makes many computable sample statistics that asymptotically approach the statistics of the underlying processes at different speeds. Limited to small-scale biochemical reaction systems with relatively single molecular species, such as transcription systems, Gillespie is easy to operate and can be parallelized. However, it is unable to provide a joint probability distribution of the variables concerned. Matrix-based methods, such as the finite state projection algorithm (FSP) and multifinite buffers (Munsky and Khammash, 2006), which reduce the state space to calculate the precise steady-state solution and variable network probability landscape (Cao et al., 2016), either rely on matrix exponentiation or eigenvalue computation to solve the truncation problem of infinite dimensional CME systems. Therefore, it is effective for large-scale biochemical reaction systems. Convenient symmetry and faster runtimes relative to the simulation methods require feature runtimes, generally $O\left(n^{3}\right)$ , where n is the state-space size. The analysis-based method is a general method for solving the main equation and can directly solve basic ordinary differential equations. For example, the steady-state solution can be reconstructed using the relationship between the generating function and the probability density function in the main equation (Gardiner, 1985), or the convolution structured method used for the basic system can easily obtain multiple properties of the solution (Jahnke and Huisinga, 2006), and its running time is generally $O\left(n\right)$ .

Through the relevant Markov jump process, numerical simulation technology to achieve an approximate solution of the CME or an effective method to solve the CME directly is still widely open for research, and methods that have the running time, application dimension, solution accuracy, and special properties of the obtained solution are still being explored. In addition to the use of CMEs, the queuing theory has been proposed to model and solve complex biochemical reaction systems for RNA production and degradation. Recently, various stochastic models describing gene burst expression have been mapped onto several specific queuing systems (Bressloff, 2017; Fralix et al., 2023; Szavits-Nossan and Grima, 2023). Solving stochastic gene expression models using queuing theory (Szavits-Nossan and Grima, 2024) provides different viewpoints to building solutions for more complex gene expression models than those currently considered.

### 2.4 Definition of key indicators of transcriptional burst

**FIGURE 4:**
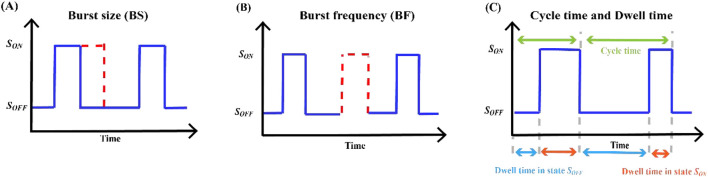
Key indicators of transcriptional burst **(A)** The burst size and the length of time the gene waits in the state $S_{ON}$ are directly proportional to mRNA production **(B)** Burst frequency is the total number of gene switches to state $S_{ON}$ per unit time **(C)** Dwell time is the length of time a gene waits in a $S_{ON}$ or $S_{OFF}$ states. The cycle period of a gene is the total length of time the gene waits in a continuous $S_{ON}$ and $S_{OFF}$ states.

RNA imaging technology has been used to directly visualize the dynamics of transcriptional bursts in cells (Larson et al., 2011). Burst measurements can help capture dynamic processes overlooked in standard population-averaged measurements of gene product expression, reflecting the underlying mechanisms of transcriptional regulation. Transcriptional burst models of gene expression can be characterized by several random variables, namely, burst size, burst frequency, dwell time, cycle time, and average travel ratio, and several studies have focused on the regulation of these parameters (Ochiai et al., 2020). Our mathematical analysis focused on building expressions for parameters based on explicable biological principles, the calculation of which is closely related to the rate of state switching, and then calculating the probability density function (PDF) or the probability mass function (PMF) of these random variables and their statistics. Basic information regarding these outbreak parameters is summarized in Table 5 and Figure 4.

**TABLE 5:** The molecular regulatory mechanisms affecting transcriptional bursts in higher eukaryotic genes correspond to those described in Figure 4.

Parameters	Definition	Formula	Function	Ref.
Burst size (BS)	The number of copies transferred by a transcription burst	$BS=\frac{s}{k_{\text{off}}}$	The randomness of gene expression is mainly determined by the explosive nature of transcription	Luo et al. (2022b), Wang et al. (2024)
Burst frequency (BF)	The number of transcriptional bursts that occur in a fixed cycle	$BF=\frac{1}{\left(\tau_{off}\right)+\left(\tau_{on}\right)}$	Understanding the temporal dynamics of gene expression and how cells quickly adapt to environmental changes	Friedman et al. (2006), Kim and Marioni (2013), Luo et al. (2022b)
		$BF=k_{off}$		
Dwell time (DT)	The sum of dwell times in total states in a single burst	$CT=\sum \tau_{S_{i}}$	Understand the dynamic nature of gene regulation	Zopf et al. (2013), Wang et al. (2024)
Cycle time (CT)	The duration of the RNA polymerase remaining on the gene for transcription	$E\left[DT_{\text{ON}}\right]=\frac{1}{\lambda_{\text{on}1}}+\frac{\lambda_{\text{off}2}}{\lambda_{\text{on}1}\lambda_{\text{on}2}}$	Reveal the efficiency of transcription processes and the overall dynamics of gene expression	Zopf et al. (2013), Donovan et al. (2018), Wang et al. (2024)
Average travel ratio (MTR)	Pol II density between genosome and promoter approximation	$E\left[TR\right]=\frac{P_{GB}}{P_{PP}}=\frac{E\left[BS\right]}{E\left[DT_{\text{rel}}\right]}\tau_{E}=\lambda_{\text{rel}}\tau_{E}$	Reveal the balance between transcriptional initiation and elongation	Wang et al. (2024)

**FIGURE 5:**
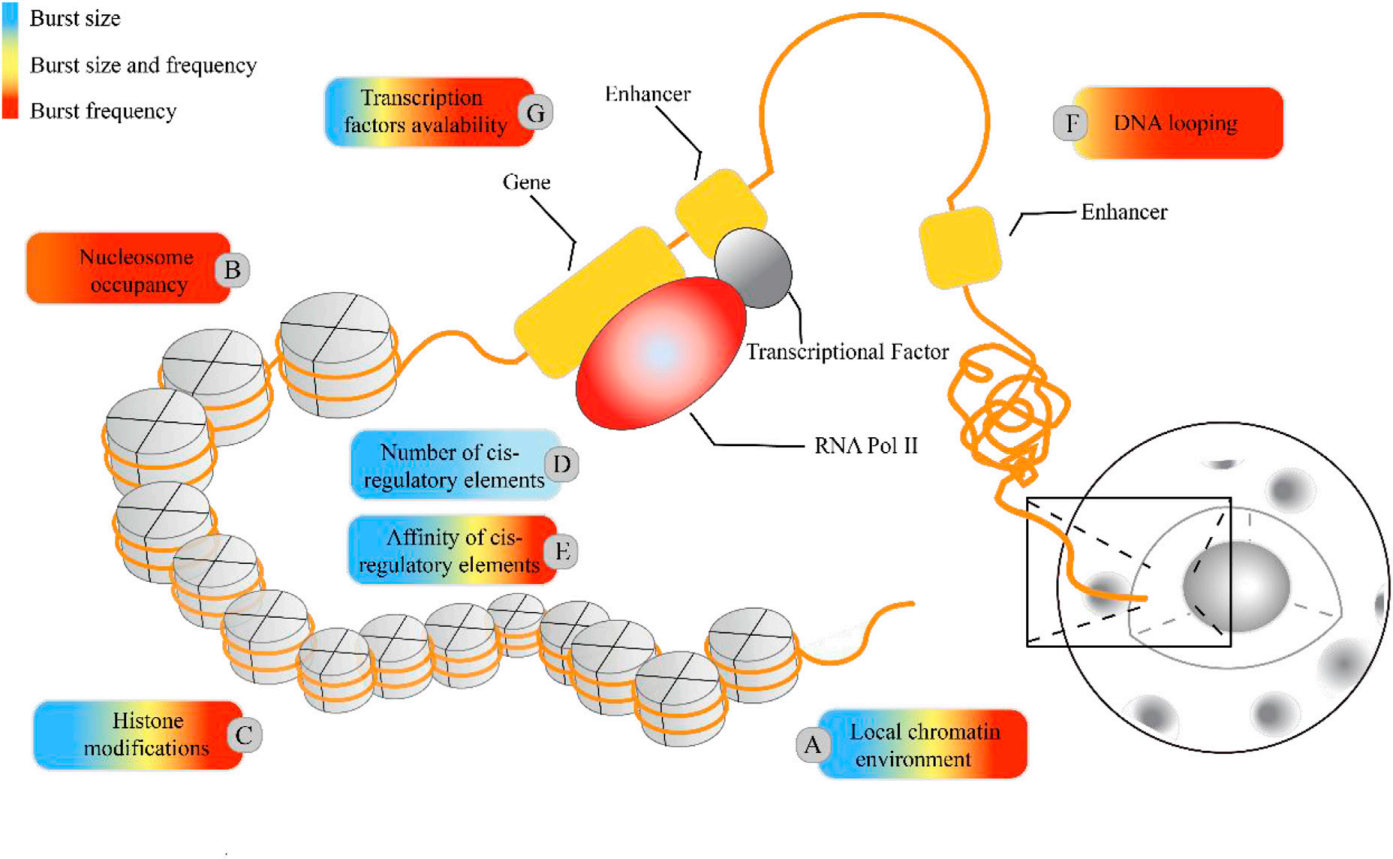
Regulatory molecular mechanisms underlying the transcriptional burst mechanism. In higher eukaryotic systems, the local chromatin environment, nuclear occupancy, histone modifications, and the number and affinity of cis-regulatory elements influence transcriptional bursting, adding to the regulatory complexity. The colored boxes highlight specific molecular mechanisms for their regulatory impact on burst size and frequency: blue represents regulation of burst size, red indicates regulation of burst frequency, and yellow indicates control over both burst size and frequency. The proportion of colors corresponds to the frequency of citations in Table 4.

#### 2.4.1 Burst size

In our characterization of transcriptional burst dynamics, burst size and burst frequency were the two most critical parameters. Transcriptome-wide data reveal that burst frequency is primarily determined by enhancers, and burst size is primarily determined by core promoters (Wang et al., 2024). Constrained by the biocentric rule (Jia et al., 2019), gene expression includes three stages: transcription, translation, and promoter switching between the active and inactive states. Based on the two-state model, mRNA synthesis occurs in random bursts and exhibits a geometric distribution. The average number of transcripts produced by a gene at each burst is called the burst size (see Figure 4A).

#### 2.4.2 Burst frequency

Based on the above model, there are two common descriptions of burst frequency. One is defined as the average number of bursts occurring per unit time (Luo et al., 2022b), that is, the reciprocal of the average cycle time, which can also be understood as the number of transcriptional bursts occurring in a fixed cycle (Friedman et al., 2006), where $\tau_{off}$ is the waiting time of the promoter in the OFF state, $\tau_{on}$ is the waiting time of the promoter in the ON state, and <⋅> is the average waiting time. Another definition is based on the rate at which the promoter state switches (Figure 4B) (Kim and Marioni, 2013).

#### 2.4.3 Cycle time

The cycle time of a gene transcriptional burst consists of two processes (Zopf et al., 2013): (i) residence time from the inactive state $S_{OFF}$ to the activated state $S_{ON}$ and (ii) dwell time from the active state to the inactive state. For different gene expression models, the cycle period is a random variable equal to the sum of the residence times of all states $S_{i}$ in an outbreak process (see Figure 4C) (Wang et al., 2024).

#### 2.4.4 Dwell time

Based on the four-state model of gene expression (Wang et al., 2024), dwell time is a random variable of the waiting time of a single state of a gene during the transcriptional burst cycle (Figure 4C). We temporarily ignored the dwell time in the $S_{OFF}$ state and calculated it in the $S_{ON}$ state (Zopf et al., 2013). Subsequently, the residence time in the $S_{ON}$ state can be calculated separately (Donovan et al., 2018; Wang et al., 2024). Let $H_{T|BS,S}\left(t\mathrm{middle}|m,s\right)$ be the survival probability of mRNAs generated in a burst (i.e., BS=m,m=0,1,2,… .) at time t (i.e., T=t ) and S state (i.e., $S=s,s\in \{\left.rec,rel\right\}$ ). According to the concept of burst size, the marginal probability of the survival time T is

$$H_{T|BS,S}\left(t|m,s\right)=\mathrm{Pr}\left(T_{exit}>t|BS=m,S=s\right),$$ (10)

where $T_{exit}$ denotes the exit time from the S state in Equation 10. For the detailed solution process in (Wang et al., 2024), the average ON-state dwell time $E\left[DT_{ON}\right]$ can be expressed as Equation 11

$$E\left[DT_{ON}\right]=\int_{0}^{+\infty }tp_{ON}\left(t\right)dt=\frac{\lambda_{rel}+\lambda_{off1}^{rel}}{\lambda_{rec}\lambda_{off1}^{rel}+\left(\lambda_{rel}+\lambda_{off1}^{rel}\right)\lambda_{off1}^{rel}}+\frac{\lambda_{rec}}{\lambda_{rec}\lambda_{off1}^{rel}+\left(\lambda_{rel}+\lambda_{off1}^{rel}\right)\lambda_{off1}^{rel}},$$ (11)

where $\lambda_{c},c\in \{\left.rec,rel,off1\right\}$ is the transfer rate of the burst process and $S_{c}$ represents each state in the model. $DT_{ON}$ is the dwell time of the ON state and $P_{ON}\left(t\right)$ is the total transition probability density function of the dwell time of the ON state. The first term on the right side of the equation is the average dwell time $E\left[DT_{rec}\right]$ of the fundraising state of Pol II, and the second term is the average dwell time $E\left[DT_{rel}\right]$ of the fundraising state of Pol II in the ON state. Similarly, the average dwell time in the OFF state is calculated as follows in Equation 12:

$$E\left[DT_{ON}\right]=\frac{1}{\lambda_{on1}}+\frac{\lambda_{off2}}{\lambda_{on1}\lambda_{on2}}\;.$$ (12)

#### 2.4.5 Average travel ratio

Based on an important characteristic parameter mentioned in the four-state model, the average travel ratio (MTR) is defined as the ratio of the Pol II concentration in the gene body (i.e., the DNA sequence of the entire region of the gene, except for the regulatory region, from the transcription of the gene start site to the polyadenylation signal) to the Pol II concentration at the proximal promoter seeing Equation 13.

$$E\left[TR\right]=\frac{P_{GB}}{P_{PP}}=\frac{E\left[BS\right]}{E\left[DT_{rel}\right]}\tau =\lambda_{rel}\tau ,$$ (13)

where τ is a fixed extension time interval of mRNA and $P_{GB}$ is the proportion of the total extension time of Pol II to the total circulation time in the genome. $P_{PP}$ is the Pol II concentration near the promoter, which can be viewed as the proportion of the residence time of the $S_{rel}$ (Pol II suspended release state) state throughout the transcriptional burst; $\lambda_{rel}$ is the effective rate of Pol II pause release (Wang et al., 2024).

### 2.5 Parameter inference methods

In a single cell, highly variable patterns of gene expression often make the production of gene expression products (mRNA and proteins) explosive. Therefore, several methods have emerged to infer the parameters of transcriptional burst dynamics from single-cell data. According to the given real gene expression data (See it in section 2.6), we first need to carry out outlier processing and normalization of the data. Secondly, we defined the transcription parameters and the master equation to solve the steady state distribution of mRNA according to the selected model (See it in section 2.1; section 2.3; section 2.4). Thirdly, we construct the state transition matrix according to the requirement of the likelihood function and select the parameter inference method. one is based on likelihood and moment; the other is the simulation-based approach (See it in section 2.5). Fourthly, you implement parameter inference and optimize the inferred parameter set. Finally, we perform parameter validation and model evaluation, including checking the simulation results with inferred parameters and fitting to real data, and evaluating the uncertainty of parameters. The relatively mature estimation methods of transcriptional burst characteristic parameters can be mainly divided into two categories: one is based on likelihood and moment and the other is a simulation-based approach.

For estimation methods based on likelihood and moment, parameters can be estimated by either explicitly calculating the likelihood of the observed data for given parameters or by comparing the moments (mean, variance, skewness, etc.) of the distribution of gene expression products (mRNA and proteins) with those predicted by the model. Typical methods include maximum likelihood estimation (MLE) and the method of moments estimation (MME), such as the general method of moments and binomial moment estimation. Daigle et al. used an iterative, simulation-based Monte Carlo expectation-maximization algorithm (modified cross-entropy Monte Carlo expectation maximization, $MCEM^{2}$ ) to compute the likelihood function for parameter estimation (Daigle et al., 2015). The advantage of this method lies in its effective estimation of parameters for stochastic biochemical systems from given incomplete data and in inferring promoter state numbers and structures. This method can be used to infer the burst size follows the geometric distribution. The MLE method maximizes the probability of observing the gene expression data given the parameters, making it a universal parameter estimation method. However, the consistency and efficiency of MLE estimates depend on large sample sizes, which make parameter optimization challenging. Moreover, MLE does not provide a natural measure of parameter uncertainty.

The hierarchical Bayesian method, which integrates prior knowledge based on Bayesian principles, is a promising parameter estimation approach. Kim et al. were the first to study the dynamics of stochastic gene expression using scRNA-seq data (Kim and Marioni, 2013). They constructed a beta-Poisson model based on a stochastic telegraph model of gene expression. The hierarchical Bayesian method assumes a gamma distribution for each gene-specific parameter in the beta-Poisson model (normalized by the degradation rate). The gamma distribution is advantageous in that it is strictly positive, possesses a simple functional form, and allows independent adjustments of its mean and variance. Finally, the hierarchical Bayesian method is combined with collapsed Gibbs sampling for parameter estimation. More importantly, it necessitates a clear computational form of the likelihood function. Sensitivity to outliers in the data can lead to numerical instability in the MLE, which is highly sensitive to model assumptions. As an alternative to the MLE method, the MME is based on the first three moments of raw gene expression counts and can more directly reflect the characteristics of the data. Larsson et al. were among the first to propose an MME method suitable for estimating the parameters of the telegraph model based on single-cell transcriptomic data (Waterborg, 1993; Ezer et al., 2016). This method utilizes the first three moments $M_{1}^{i},M_{2}^{i},M_{3}^{i}$ from each gene. With the advancement of single-cell sequencing technology, the exponential moments for estimation combined with scRNA-seq data are represented as follows in Equation 14:

$$M_{1}^{i}=\frac{1}{Q}\sum_{j=1}^{Q}x_{ij},$$

$$M_{2}^{i}=\frac{1}{Q}\sum_{j=1}^{Q}x_{ij}\left(x_{ij}-1\right),$$

$$M_{3}^{i}=\frac{1}{Q}\sum_{j=1}^{Q}x_{ij}\left(x_{ij}-1\right)\left(x_{ij}-2\right).$$ (14)

The parameters for each gene are estimated separately according to the continuous ratio of the exponential moments as Equation 15:

$$r_{1}=M_{1},r_{2}=M_{2}/M_{1},r_{3}=M_{3}/M_{2}.$$ (15)

The estimated kinetic parameters are like Equation 16

$$k_{on}=\frac{{2r}_{1}\left(r_{3}-r_{2}\right)}{r_{1}r_{2}-2r_{1}r_{3}+r_{2}r_{3}},$$

$$k_{off}=\frac{{2r}_{1}\left(r_{3}-r_{2}\right)\left(r_{1}-r_{3}\right)\left(r_{2}-r_{1}\right)}{\left(r_{1}r_{2}-2r_{1}r_{3}+r_{2}r_{3}\right)\left(r_{1}-2r_{2}+r_{3}\right)},$$

$$k_{syn}=\frac{3r_{1}r_{3}-2r_{1}r_{2}+r_{2}r_{3}}{r_{1}-2r_{2}+r_{3}}.$$ (16)

Although the MME overcomes the limitations of the sample size, the relationship between moments and parameters is not always consistent, which may lead to biased estimates. Furthermore, the MME is sensitive to the data distribution, particularly the behavior of the tails (e.g., heavy-tailed data distributions). Skewed distributions or outliers can affect the sample moments, ultimately affecting the estimation results.

For simulation-based estimation methods, the parameters can be estimated by minimizing the distance between the model distribution and the observed data. Typical approaches include those based on the Bayesian theory, such as approximate Bayesian computations (ABC) and neural network techniques. These methods do not rely directly on the computation of the likelihood function but instead approximate or infer the posterior distribution of key parameters for transcriptional bursts through extensive simulations. Toni et al. applied ABC methods based on sequential Monte Carlo (SMC) for parameter estimation and model selection in dynamic models (Toni et al., 2008). For simulation-based estimation methods, parameters can be estimated by minimizing the distance between the model distribution and the observed data. Typical approaches include those based on the Bayesian theory, such as ABC and neural network techniques.

Machine learning and deep learning methods have developed rapidly in recent years. Jiang et al. developed an artificial neural network (ANN) with a universal function approximator to study the non-Markov models of gene expression and transcriptional feedback (Jiang et al., 2021). The principle involves approximating non-Markov models with simpler stochastic models using ANNs. They utilized ANNs in conjunction with the maximum likelihood approach to infer sets of transcriptional dynamic parameters (including gene activation and inactivation rates, transcription rates, burst frequencies, and burst sizes) from the synthetic data. The ANN method of must be solved using a finite state projection algorithm (FSP). For multispecies interaction systems in scRNA-seq data, a universal closed-form solution for the CME has not yet been developed, and challenges remain in computing biophysical parameters. Recently, within a CME system encompassing transcriptional bursts, splicing, and degradation, Gorin et al. proposed a kernel-weighted regression (KWR) method that requires learning with neural networks (Gorin et al., 2022). This method represents a multidimensional solution of the CME for simulating transcriptional dynamics, specifically, the steady-state joint distribution solutions of the two models that approximate the RNA lifecycle. The authors integrated both KWR and Parameter Scaling KWR (psKWR) neural approximation strategies into a maximum likelihood estimation framework to infer sets of gene expression parameters in mouse brain cells.

In the future, time-resolved single-cell data with spatial information will become the primary focus for constructing mathematical models to study the dynamics of transcriptional bursting. The challenge of combining more flexible statistical methods to minimize the impact of noise and thereby infer parameters related to transcriptional bursting remains to be addressed (Luo et al., 2022a).

### 2.6 Approaches to studying data-driven dynamics of transcriptional burst

The data-driven construction of single-cell gene expression dynamics has been the preferred approach in several studies (Jovic et al., 2022). The rapid advancement of single-cell sequencing technologies in recent years has provided extensive data for individual analyses and multiomics studies to elucidate gene expression features and regulatory mechanisms at the single-cell level. The results obtained from single-cell sequencing data enable not only transcriptomic-level analyses but also the exploration of genomic and epigenomic heterogeneity fluctuations within cell populations (Kashima et al., 2020). As the demand for broader applications and increased precision in omics feature analysis of single-cell sequencing data has grown within the field, the concept and technology for integrating multilevel single-cell sequencing data have gradually taken shape and developed. However, the lack of spatial information remains a limitation of single-cell sequencing technology. Spatial transcriptomics, which has recently been widely discussed, can identify differential expression patterns according to local environmental conditions within tissues (Kumar and Manning, 2022). Integrating temporal and spatial information to deeply dissect omics features at various levels within each cell and comparing results from integrated data and imaging techniques with tissue pathology provide new insights into the mechanisms of transcriptional bursting in gene expression (Kashima et al., 2020).

#### 2.6.1 Studies using only single-cell transcriptome sequencing data

To probe cell identity, status, function, and response, scRNA-seq is an alternative method for analyzing gene expression activity in cells. ScRNA-seq is a whole-genome sequencing method that extracts dynamic behavior from static measurement distributions. It allows single-cell-level transcriptomes of millions of cells to be analyzed in a single experiment to classify, characterize, and distinguish individual cells, thereby identifying populations of cells that are few in number but are significantly functional (Jovic et al., 2022). Therefore, scRNA-seq can uncover low-abundance but critical features of rare cells that are often masked by vast dominant expression signals, thereby enhancing the utility of single-cell transcriptomic sequencing data (Tang et al., 2009; Armingol et al., 2020). In 2009, Tang et al. reported a more mature scRNA-seq technology for generating high-throughput transcriptomic data (Tang et al., 2009; Rodriguez and Larson, 2020). When analyzing the burst characteristics and regulatory mechanisms of gene expression implicit in scRNA-seq, it is necessary to make appropriate assumptions about gene expression mechanisms when building mathematical models. These assumptions, such as the limiting rate and step of promoter state switching, are necessary even if they affect the model’s accuracy. Although scRNA-seq is widely used in high-throughput sequencing assays, it usually only measures mature RNA abundance, which is determined by both RNA synthesis and degradation (Blumberg et al., 2021). Therefore, a more direct approach to understanding transcriptional dynamics is to leverage nascent RNA sequencing techniques (NRS), which can directly capture active RNA polymerases in the nuclei, such as PRO-seq (Mahat et al., 2016). Recently, Zhao et al. developed a statistical model to estimate transcription rates for NRS data; however, estimating parameters for transcription bursts is challenging if the data are not at the single-cell level (Zhao et al., 2023). Fortunately, single-cell NRS recently has been developed (Mahat et al., 2024), allowing direct estimation of burst size and frequency. Therefore, descriptive results from data-driven and phenomenological analyses alone are insufficient to explain this biomechanism. Only by combining single-cell data with a statistical physical model can we accurately, robustly, and flexibly infer burst dynamics and reveal the biophysical mechanisms of gene regulation.

#### 2.6.2 Studies using integrated data

Although scRNA-seq data have been widely used in multiple fields such as immunology, developmental biology, and oncology, multidimensional data generated by single-cell sequencing are sparse and do not provide complete information about protein levels or post-translational modifications. Therefore, the selection of appropriate tools for computational analysis according to the research context and datasets is necessary. The study of spatial omics data and the integrated use of multiple-omics data will push single-cell technology into a wider range of scientific and translational research, expanding the scope for health monitoring, disease diagnosis, and in-depth analysis of genomic, epigenomic, and transcriptomic data characteristics (Tunnacliffe and Chubb, 2020).

The mechanisms of gene expression regulation have been studied using sequencing. To investigate the role of epigenomic data in transcription dynamics (Kashima et al., 2020; Ma et al., 2022), protein-DNA interactions can be directly detected using single-cell chromatin immunoprecipitation and sequencing (scChIPseq) from the perspective of the local chromatin environment, nucleosome occupancy, histone modification, and number and affinity of regulatory elements (transcription factors). This, in turn, helps identify protein-binding sites for genes of interest, such as transcription factor-binding sites and chromatin tissue heterogeneity. ScATAC-seq, which studies open chromatin from the perspective of the local chromatin environment and assists in accessing genome-wide chromatin, can help determine cell types at a single-cell resolution, analyze intercellular heterogeneity, and identify many different modes of gene regulation (Kashima et al., 2020; Wu et al., 2021). From the perspective of the DNA dissociation rate, transcription is a discontinuous process in eukaryotes, and mRNA is produced explosively after transcription factors bind to regulatory elements in the genome (Mazzocca et al., 2021). To investigate the role of genomic data in transcriptional dynamics, it is essential to understand the influence of transcription factors on the frequency, duration, and size of transcriptional bursts. ScSLAM-seq can visualize and explain differences in transcriptional activity at the single-cell level, describing “ON/OFF” switching in gene expression and transcriptional burst dynamics (Erhard et al., 2019). In living organisms, the structure of genetic information determines the basic properties, which in turn determine the function and use of cells. From the perspective of nuclear structure (including DNA cycling, promoter-enhancer contact, and nuclear regionalization), we can extract two main features of enhancer-promoter communication through extensive genome-wide studies (4C, 5C, and Hi-C): (1) communication between enhancers and promoters may be mediated by chromatin loops and (2) the genome is organized into topologically associating domains (TADs) that may delineate local gene activity. In addition, enhancer–promoter communication mainly regulates burst frequency rather than burst size (Daigle et al., 2015; Wang et al., 2024). In fixed cells, the application of the standard smFISH technique for single RNA imaging generated a distribution of nascent and mature mRNA counts in single cells and provided a new dataset for large-scale single-molecule studies. Conventional single-cell sequencing techniques often lack spatial information regarding cells; however, high-throughput and multiplexed datasets compensate for this limitation (Wang et al., 2018). The development of transcription-regulated live-cell imaging techniques has greatly facilitated the search for molecular mechanisms underlying precise spatiotemporal gene expression programs (Daigle et al., 2015).

Single-cell sequencing data can effectively characterize the omics of genomic, epigenomic, and transcriptomic data. Therefore, many studies have attempted to overcome these barriers of information disability by integrating single-cell sequencing data from different omics layers. On the one hand, the activity and integrity of a cell are destroyed in the process of sequencing a single omics layer for a cell, preventing the simultaneous analysis of different levels of omics information from the same cell. On the other hand, the abundance of single-cell sequencing data can improve the accuracy of cell characterization. The model of transcriptional burst dynamics using integrated data can ensure the high fidelity of accurate transcriptional regulation. Additionally, the diversity of data sources can fully characterize the randomness of biochemical reactions and reveal cellular heterogeneity (Wang et al., 2016; 2018; Kashima et al., 2020). Correlation analysis of gene features, such as burst parameters and promoter structure at the single-cell level, will help analyze the regulatory mechanism of gene expression dynamics. The development of single-cell techniques has gradually enriched the information contained in the data, allowing us to understand multiple transcription factors, their interactions, and their effects on the transcriptional output of specific target genes in the same living cell, thus providing opportunities to further understand the mechanisms of transcriptional bursts in the future.

Currently, the study of transcription and transcriptional bursts using single-cell transcriptome data is extensive. The single-cell transcriptome data discussed in the previous section and the single-cell genome sequencing data and epigenome data discussed in this section are at the same level and are independent. Therefore, an effective computing strategy is needed to determine the relationship between different levels of omics data and integrate them to approximate the multilayer sequencing results of the same cell. By obtaining prior information on different levels of omics of the same cell, insights will be more comprehensive. This approach presents an opportunity to move from descriptive “snapshot” conclusions to a deeper revelation of the mechanisms underlying cellular transcriptional bursts.

## 3 Prospect

The stochastic nature of transcriptional bursting dynamics during gene expression is an important source of phenotypic heterogeneity. Bursts and periods of silence occur alternately during the mRNA synthesis, corroborating that genes are predominantly inactive during the transcriptional bursting cycle and that the bursts are brief. The multi-state model of gene expression is based on this unique biological mechanism. Specifically, judicious selection of multi-state models of gene expression is important for the accurate analysis of real single-cell transcriptomic sequencing data. The stochastic telegraph model is a fundamental choice in gene regulation studies. However, there is a lack of comprehensive research demonstrating that models with more transcriptional states better fit the actual data. In contrast, simpler models with two, three, or four states can often predict gene expression with a lower theoretical bias.

In this study, we critically examined conventional gene expression models, explained the temporal scales and regulatory factors implicated in transcriptional bursting, and assessed the methodologies employed in gene expression research from the perspectives of biostatistical and biochemical network analysis. To uncover the regulatory underpinnings of stochastic gene expression, we focused on key factors, including burst size, frequency, cycle period, residency time, and travel ratio, to elucidate the mechanisms underlying bursting dynamics. We also categorized the foundational concepts and enumerated methods for parameter inference within a bursting dynamics framework. Finally, we explored the current landscape and identify challenges in the evolving field of transcriptional bursting dynamics propelled by advancements in single-cell sequencing data. Although the study of transcriptional bursting dynamics has advanced over the last decade, analysis of scRNA-seq and other omics data, along with cutting-edge technologies, continues to evolve. These ongoing developments promise to contribute substantially to the progress of systems biology and bioinformatics.

Spatial transcriptomics technology fills the gap in spatial distribution information that scRNA-seq sequencing technology lacks. By facilitating precise comparisons between gene expression patterns and histopathological information, it enables the identification of differential expression patterns within the local tissue microenvironment, as well as, provides insights into cell-to-cell interactions and signal transduction. One significant challenge with the advent of spatial transcriptomics is the absence of a standardized analysis workflow. Developing spatial dynamic models of cell communication or models that reflect gene regulation by integrating spatial transcriptomic data with other omics datasets remains a crucial task for gaining more comprehensive insights into computational systems biology. In the future, we should focus on the dynamics of key transcription factors and enhancers, as well as on phase separation methods, for the quantitative study of the dynamic parameters influencing bursting. Progress in single-molecule techniques will enhance these analyses, allowing imaging of multiple transcription factors, their interactions, and their impact on the transcriptional output of specific target genes within the same living cell. In the future, the development of a unified analytical workflow that combines *in vivo* imaging, single-cell sequencing, and mathematical modeling will permit systematic analysis of bursting behavior across multiple genomic loci.
